# Popular Hybrid Congenital Heart Procedures without Cardiopulmonary Bypass

**DOI:** 10.3389/fsurg.2017.00009

**Published:** 2017-03-06

**Authors:** Aamisha Gupta, Zahid Amin

**Affiliations:** ^1^Division of Pediatric Cardiology, Children’s Hospital of Georgia, Augusta University, Augusta, GA, USA

**Keywords:** hybrid, perventricular, stent, pulmonary valve, stent

## Abstract

As surgical and catheter interventions advance, patients with congenital heart disease are now offered alternative treatment options that cater to their individual needs. Furthermore, collaboration between interventional cardiologists and cardiac surgeons have led to the development of hybrid procedures, using the best techniques of each respective field to treat these complex cardiac entities from initial treatment in the pediatric patient to repeat intervention in the adult. We present a review of the increased popularity and trend in hybrid procedures in congenital heart disease without the use of cardiopulmonary bypass.

## Common and Popular Hybrid Procedures

1. Hybrid Stage I palliation for hypoplastic left heart syndrome (HLHS).
2. Perventricular ventricular septal defect (VSD) closure; muscular and perimembranous.
3. Perventricular pulmonary valve implantation.
4. Hybrid pulmonary valvuloplasty for pulmonary atresia.
5. Hybrid aortic valvuloplasty.
6. Hybrid stent placement in the branch pulmonary arteries.
7. Per-atrial atrial septal defect closure.
8. Hybrid paravalvar leak closure of the mitral valve.
9. Hybrid paravalvar leak closure of the aortic valve.
10. Hybrid procedures for stent placement in pulmonary vein for recurrent venous stenosis.
11. Hybrid stent placement in coarctation of aorta.

A detailed description of all the above procedures cannot be addressed in this manuscript. We will, however, address what we believe are the most common of the above procedures and some that may become more popular in the near future.

## Hybrid Stage I Palliation in HLHS

Patients with a “single ventricle,” whether defined anatomically or physiologically, are some of the most complex of patients. HLHS can consist of a variety of left heart anatomical combinations that lead to left ventricular and aortic underdevelopment. Traditionally, a surgical staged palliation is performed with Norwood procedure, followed by a bidirectional cavopulmonary anastomosis (bidirectional Glenn), and Fontan. However, in infants with multiple comorbidities and low birth weight, restrictions and high risks are involved with cardiopulmonary bypass (1, 2). For these reasons, a hybrid palliation procedure that can circumvent cardiopulmonary bypass and its associated complications appeared more attractive.

### Technique

In 1993, the first reported hybrid stage I approach in HLHS was described by Gibbs et al. where through a median sternotomy the branch pulmonary arteries were banded, and the patent ductus arteriosus (PDA) was percutaneously stented (3). The technique was later revised in 2002 by Akintuerk et al. (4) and later adopted by Galantowicz et al. (5) and Bacha et al. (1), where the procedure has continued to be refined with a steep learning curve.

The primary goals and procedural steps as described by Galantowicz and Cheatham are (6)

1. unobstructed systemic output *via* the ductus arteriosus,
2. balance of the pulmonary and systemic circuits, and
3. unobstructed flow *via* the atrial septum.

**Figure 1:**
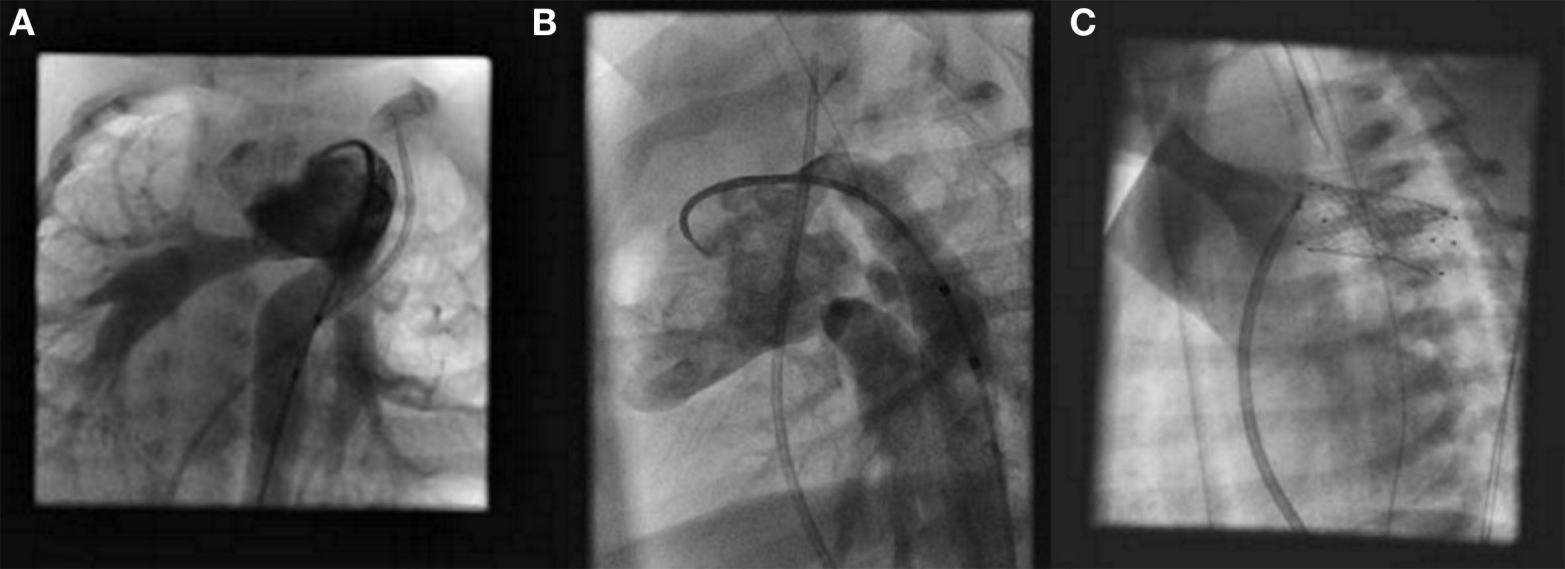
**Hybrid stage I palliation: angiography of branch pulmonary arteries after bilateral pulmonary artery banding in a right anterior oblique view (A) and left anterior oblique view (B)**. Fluoroscopic image of stent implantation within the patent ductus arteriosus **(C)**.

The patient is placed under general anesthesia, and a median sternotomy is performed. The branch pulmonary arteries are banded to a degree suitable by follow-up angiography and pressure measurements (Figures 1A,B). This step is done first to optimize cardiovascular hemodynamics in both the pulmonary and systemic circuits. Following this step, the main pulmonary artery (MPA) is directly punctured and a sheath is placed for stent delivery within the PDA (Figure 1C).

The atrial septum is addressed at a subsequent date by standard percutaneous balloon atrial septostomy, if needed, and is usually performed 1–2 weeks after the hybrid procedure when the neonate is hemodynamically stable (6).

The hybrid approach thus allows for palliation without cardiopulmonary bypass or cardiac hypothermia and therefore allows time for neonatal growth prior to the second stage. The comprehensive stage II repair is performed around 6 months of age, it is more extensive and involves PDA stent removal, aortic arch reconstruction, branch pulmonary artery de-banding with revision of the branch pulmonary arteries, along with a bidirectional cavopulmonary anastomosis (5, 6).

### Patient Selection and Outcomes

The goal of staged single ventricle palliation is to normalize the systemic ventricle volume and pressure load and maintain unobstructed cardiac output with adequate oxygen saturated blood, while also protecting the pulmonary vascular bed for subsequent palliations. Neonates present a particular challenge as their pulmonary vascular resistance is elevated early in life, requiring a staged approach to control pulmonary blood flow and allow development of the vascular bed (7). Furthermore, infants with low birth weight, prematurity, and coexisting comorbidities add increased complexity and challenges.

The optimal patient for utilization of the hybrid stage I approach has not been clearly defined as of yet. However, in a study by Karamlou et al., pediatric centers with high use of the hybrid stage I palliation were those that had greater mortality rates after the traditional Norwood procedure (8). Additionally, in that study, patients in whom the hybrid palliation was performed were indeed higher risk patients. High-risk patients are defined as infants with low birth weight <2.5 kg, prematurity, ventricular dysfunction, severely hypoplastic ascending aorta (<2 mm), genetic malformations, and severe non-cardiac comorbidities, which have been found to have as high as 60% mortality risk rates after the Norwood procedure (1, 2, 9–11). Therefore, those neonates with complex medical comorbidities or those traditionally found to have increased risk with associated initial surgical palliation have been proposed as a population set where the hybrid approach could be considered.

In patient populations with increased risk factors, there have been studies demonstrating the hybrid approach as a valuable alternative. Venugopal et al. reported 14 of 21 patients surviving the initial procedure, to later go on to comprehensive stage II palliation (2). Bacha et al. reported a 78.5% hospital survival rate after the initial hybrid palliation with 8 of their initial 14 high-risk patients undergoing stage II repair (1). Furthermore, when compared to the Norwood procedure, there are similar results to survival after Fontan completion with overall survival of approximately 80% (5, 12, 13). Alternatively, the approach has also been shown to be a beneficial tactic in biventricular repair for patients with borderline small left heart structures (14). Therefore, these studies demonstrate that both the traditional surgical approach and the hybrid approach have shown to provide adequate physiology for Fontan completion in single ventricle palliation. However, of note are the technical considerations with the hybrid procedure. There has been reported need for increased pulmonary artery re-intervention, ductal stent re-intervention, or the development of preductal retrograde coarctation (1, 5, 12, 15–17). Nevertheless, as experience expands, and the technique continues to be refined, this approach may offer a replaced standard for those complex infants where increased surgical jeopardy is a concern.

## Hybrid VSD Closure

The ventricular septum is highly complex and consists of an inflow, trabecular/apical, and outflow region; it has two separate tissue characteristics: a muscular and membranous portion, and is surrounded by the different morphological structures of the right ventricles (RVs) and the left ventricles (LVs). Muscular VSDs can be described as central, apical, anterior, or posterior. VSDs can be malaligned, committed to one or both outflow tracts, and can exist as an isolated defect, multiple defects, and/or associated with other congenital cardiac lesions (18–20).

Traditionally, large muscular and perimembranous ventricular defects were closed *via* patch repair with surgery requiring cardiopulmonary bypass, with closure at times challenging due to location and periodically resulting in residual defects (21). The percutaneous approach with device closure was first described in 1988 (22), and with refinement over the years, has provided benefit in suitable cases. However, the percutaneous approach is also technically challenging, as limitation in catheter manipulation in small babies prevents closure of complicated defects. Additionally, the delivery sheath and its sizes are never too soft for smaller babies. This increases the risks of vascular injury, as well as possible adverse events such as hemodynamic compromise and arrhythmias (23).

With the goal to avoid the risks associated with prolonged cardiopulmonary bypass, the first perventricular closure of a muscular VSD was performed in a baby by Amin et al. in 1998 (24), followed by perventricular closure by Amin et al. in 2004 of a perimembranous VSD (25). Since then, the hybrid approach has been shown to be an acceptable alternative, with minimal incision, relatively straight access to the defect, avoidance of cardiopulmonary bypass during VSD closure, and the potential to both small and large patients without the risk of hemodynamic compromise.

### Technique

For the hybrid approach, transesophageal (TEE), or occasionally epicardial, echocardiography is performed to assess the defect, guide during device placement, and assess after device release. There have been descriptions of the perventricular or per-atrial approach through a median sternotomy, a modified partial median sternotomy, or a left anterior parasternal incision at the third intercostal space (in cases of supracristal VSDs) (26).

**Figure 2:**
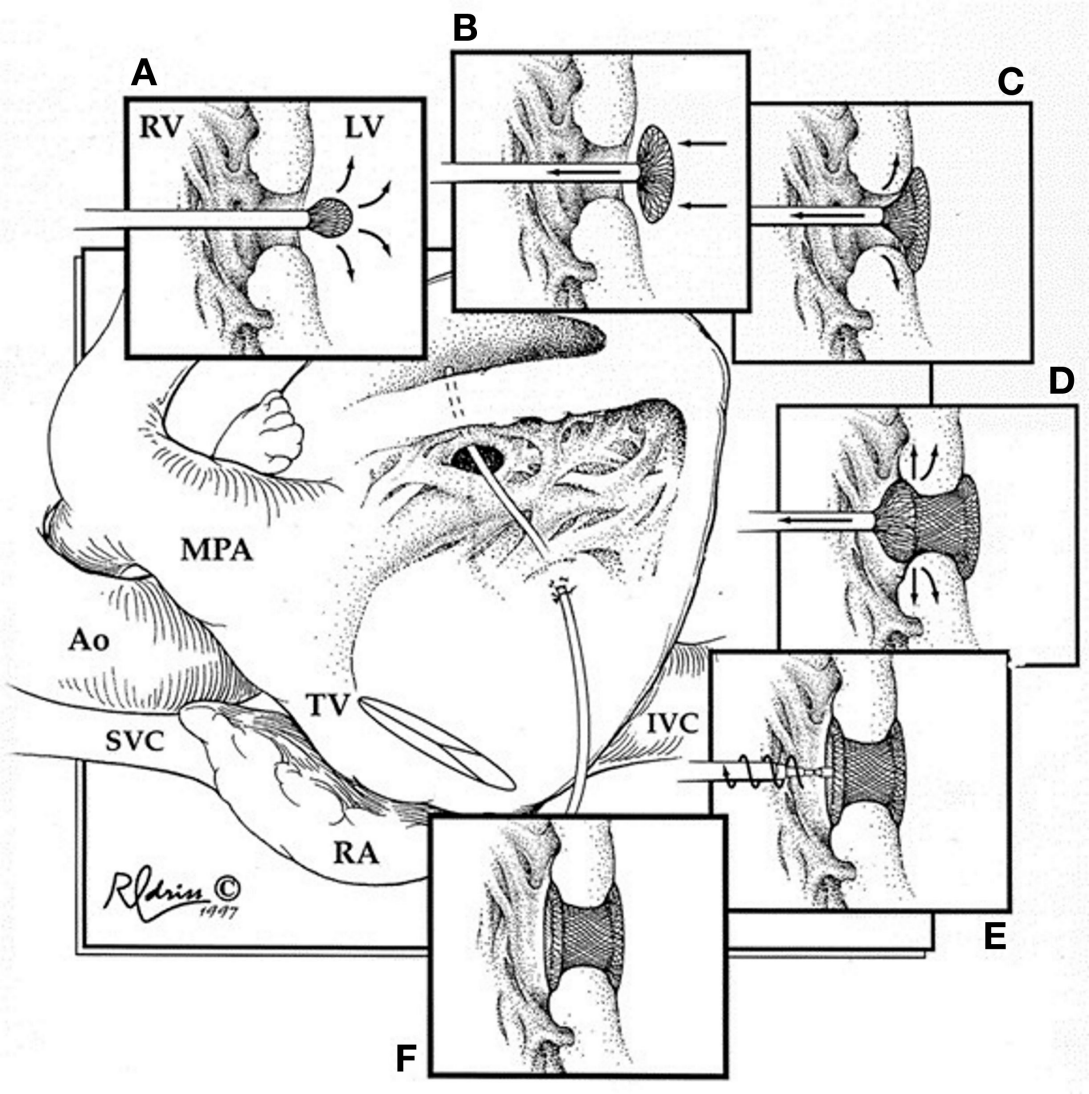
**Hybrid muscular ventricular septal defect closure**. Schematic diagram illustrating the steps involved in deployment of the device through the right ventricular free wall. RV, right ventricle; LV, left ventricle; MPA, main pulmonary artery; Ao, aorta; SVC, superior vena cava; TV, tricuspid valve; RA, right atrium; IVC, inferior vena cava. *Reprinted with permission from publisher: Elsevier* (24).

**Figure 3:**
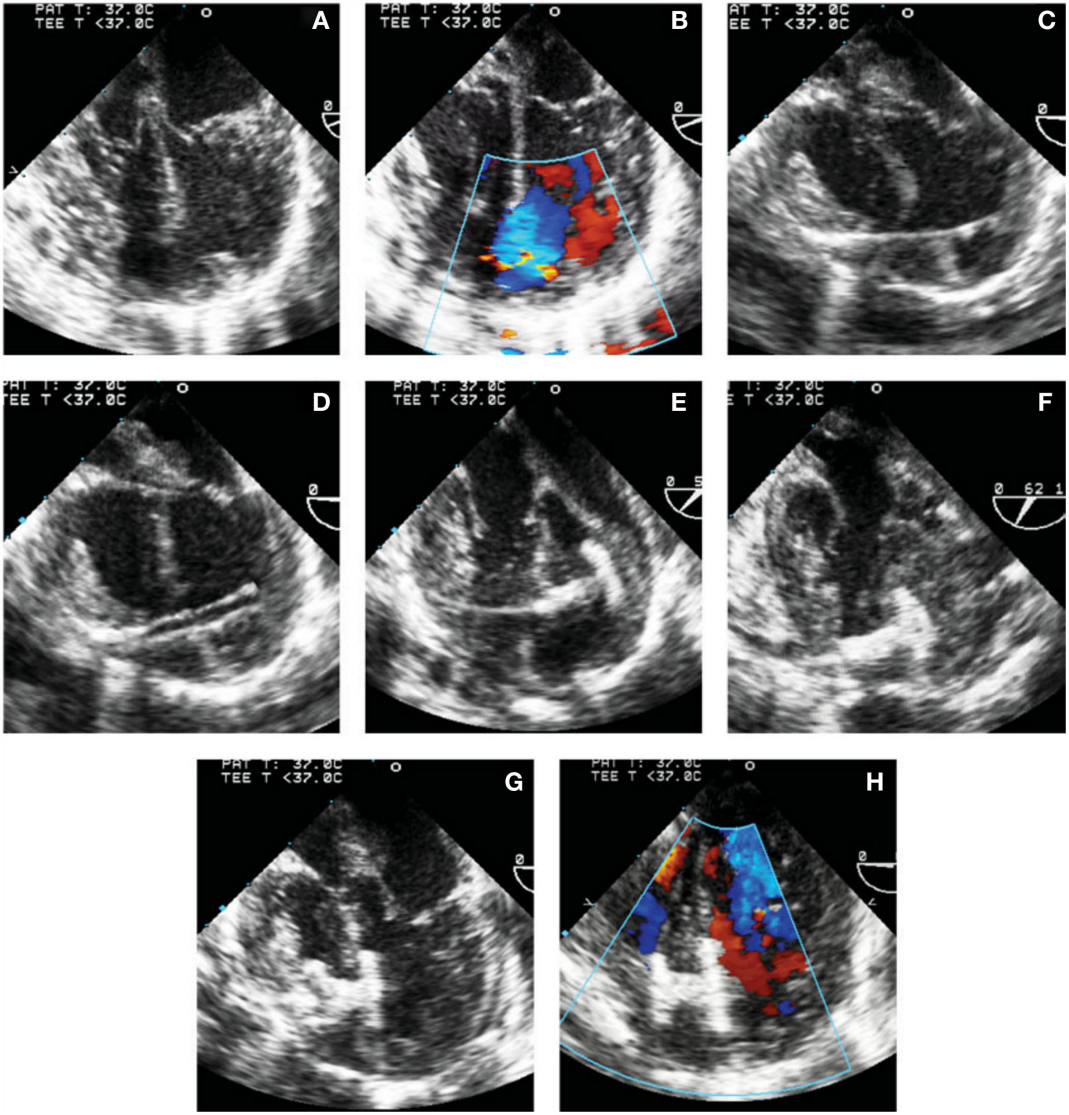
**Transesophageal echocardiographic images of steps involved in perventricular ventricular septal defect (VSD) closure**. **(A,B)** 4-chamber view without **(A)** and with **(B)** color Doppler showing the mid muscular VSD. **(C)** Wire through the right ventricular free wall and across the defect. **(D)** Delivery sheath in the left ventricle (LV) cavity. **(E)** Left disk deployed in the LV. **(F)** Left disk aligned with the ventricular septum. **(G)** Device waist and part of the right disk have been deployed. **(H)** Complete deployment of the right disk with optimal device position. *Reprinted with permission from publisher: John Wiley and Sons* (29).

During this technique, the RV free wall is exposed, and the puncture site is determined by pressing on the RV free wall while assessing by TEE, to ensure the site is as perpendicular to the VSD as possible. Then, purse string or pledgetted mattress sutures are placed around the puncture site, and a wire is introduced *via* the angiocatheter across the VSD, and the wire is exchanged for the delivery sheath, with or without serial dilation. The device of choice is loaded under echocardiography, the left disk is deployed, and while retracting the sheath slowly, the waist is deployed within the defect itself, followed by deployment of the right disk within the RV (Figure 2). Prior to release, thorough evaluation with echocardiography is performed to ensure proper placement and no residual defects (Figure 3). The device is released followed by sheath removal, and the purse string sutures are tied to close the RV puncture site. The chest is closed in routine fashion.

### Devices

There have been various devices developed and used for the closure of VSDs with some out of fashion and some still on the bench for experimentation. Presently, the most popular devices used are the Amplatzer VSD Occluders (AGA Medical, Plymouth, MN, USA) (27–29). Off label use of Amplatzer duct occluders is also relatively common (30). Initial and midterm studies show promising results (31).

### Use in Different VSD Types

The indications for perventricular perimembranous VSD closure are generally the same as the surgical indications. Of note, perimembranous VSDs pose a challenge to any type of repair, given their location adjacent to the aortic valve, mitral and tricuspid valves (TVs), and electrophysiological circuit. In a review by Yin et al., of the reported cases from 2008 to 2013, the perventricular complete closure success rate was 77–100%, and higher than that of muscular VSDs (26). Additionally, the incidence of complete heart block was lower than the percutaneous approach, 0.6 versus 2%, respectively (31). Another possible complication reported is valve regurgitation, with device-induced tricuspid regurgitation being the most common, followed by aortic valve regurgitation, most resolving or asymptomatic at follow-up (31).

Muscular VSDs are the second most common subtype of VSDs (19). Apical and anterior muscular VSDs can be difficult to access *via* a surgical trans-atrial approach and the right ventriculotomy approach due to limited view through the RV trabeculations. Additionally, left ventriculotomy has been associated with ventricular dysfunction, arrhythmia, and aneurysms (32). In a recent review of reported studies from 2003 to 2013, the reported success rate of perventricular device complete closure of muscular VSDs is 50–100% with no reported case of RV dysfunction, lower rates of arrhythmias when compared to surgical closure, and 80% closure rate at midterm follow-up (though the review admits to limitations with small study sizes in the reports) (26). In a long-term follow-up by Kang et al., at a median time of 6.5 years post device implantation, of 10 patients who underwent the technique, 5 had complete closure, and 4 patients had residual hemodynamically insignificant defects. One patient had TV entrapment from the device and underwent surgical removal and repair (33). Therefore, as longer term results are generated, the benefit of perventricular muscular VSDs can be further evaluated and may provide an acceptable alternative to surgical repair.

## Hybrid Pulmonary Valve Interventions

Right ventricular outflow tract (RVOT) structural and congenital heart disease with obstruction in the form of pulmonary valve stenosis or atresia have previously been treated surgically with transannular patch repair/RVOT conduit or valvotomy, or percutaneously with transcatheter pulmonary valve/RVOT stent or balloon valvuloplasty. The palliations lead to either restenosis or pulmonary regurgitation; this may lead to RV hypertrophy or dilation, further progressing to ventricular dysfunction and arrhythmias. There is no consensus regarding the timing of pulmonary valve replacement (34). However, in patients where weight, complex comorbidities, vascular access, venous occlusion, previous multiple sternotomies, or risk of cardiopulmonary bypass are limitations and pose increased risk for complication, there are new and innovative hybrid approaches that have been developed. And, in this anatomical scenario, the hybrid procedure allows for intervention in a range of patients, from pediatric to adult.

### Perventricular Transcatheter Pulmonary Valves

Transcatheter pulmonary valve replacements have been steadily increasing since the first transcatheter pulmonary valve replacement in 2000 (35, 36). Since then, there are new innovations in the development and use of transcatheter pulmonary valves. Yet, presently, there are two common transcatheter pulmonary valves being used in the USA as described by a recent review (35). The Melody valve (Medtronic, Minneapolis, MN, USA) is a bovine jugular venous valve that is available in the following diameters: 18, 20, and 22 mm. The Edwards Sapien (Edwards Lifesciences LLC, Irvine, CA, USA) valve was originally conceived for aortic valve replacement but was then successfully used in the pulmonary position (37). This is a bovine pericardial valve mounted within a stainless steel stent, with diameters of 23 and 26 mm. The offspring of the Edwards Sapien valve is the newly developed and FDA-approved Sapien XT (Edwards Lifesciences LLC, Irvine, CA, USA) with diameters of 23, 26, and 29 mm, but only requiring an 18-19-Fr introducer.

The inclusion criteria for percutaneous transcatheter pulmonary valve placement were patients >30 kg and >5 years of age, likely to reduce the risk of vascular injury due to the large size of the 22-Fr delivery sheath for Melody valve placement (38, 39). However, recently Berman et al. (40) reported 25 patients, median weight 21.4 kg, where percutaneous Melody valve placement was successful in 23 patients. However, there still lies the risk in the population subset where venous access size or occlusion poses an issue to traditional percutaneous sheath placement.

The hybrid pulmonary valve approach was first described as a bailout technique following failed percutaneous delivery in patients at risk for cardiopulmonary bypass. Simpson et al., Berman et al., and Cubeddu and Hijazi described the technique as a bailout approach in larger patients, ages 13, 16, and 44 years with both the Melody and Edwards Sapien valve (41–43). In these case reports, the transcatheter valve could not be successfully placed percutaneously, due to angle, malposition, or embolization into the RVOT. Therefore, after successful surgical removal of the failed valve, transcatheter pulmonary valves were placed *via* the perventricular approach. The hybrid technique was subsequently performed electively in a 12-kg patient (44).

### Technique

Prior to the hybrid procedure, a routine diagnostic catheterization is performed *via* the traditional femoral approach with angiography of the RVOT, MPA, and branch pulmonary arteries. Coronary artery compression testing with balloon inflation of the RVOT must be done either during the diagnostic catheterization or during the hybrid procedure, prior to transcatheter valve placement, to ensure safe distance of the coronaries.

**Figure 4:**
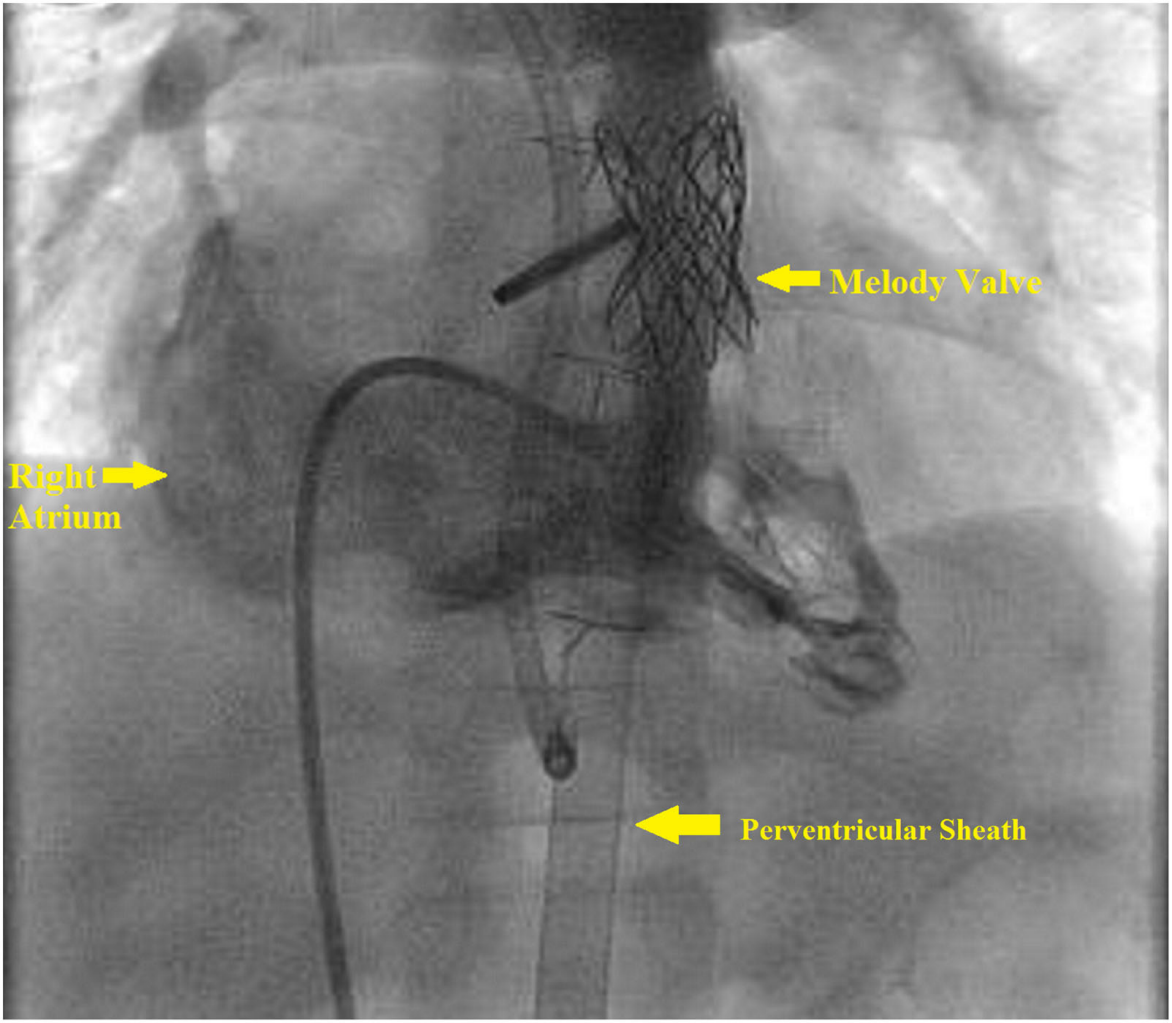
**Right ventricle (RV) angiogram**. Angiographic catheter advanced antegrade *via* femoral sheath and into RV and demonstrates Melody valve in proper position.

**Figure 5:**
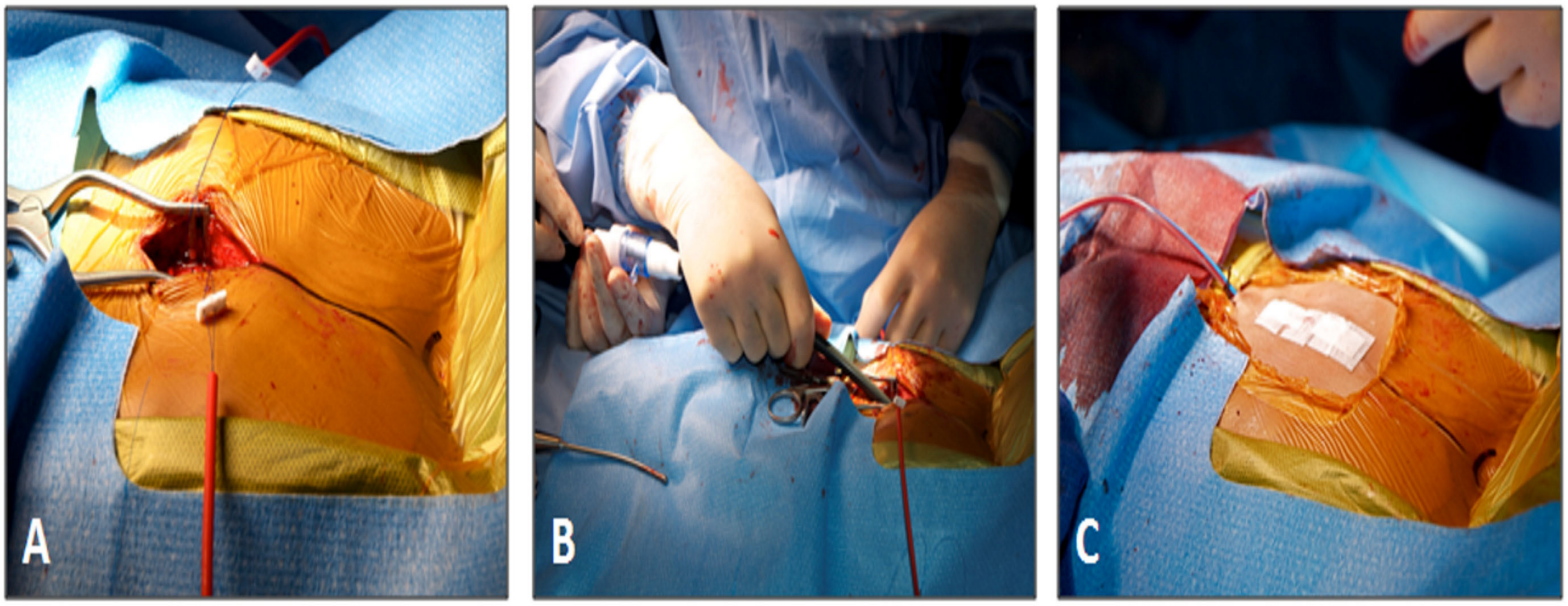
**Subxiphoid access for perventricular Melody valve implantation**. **(A)** The size and location of the surgical incision. **(B)** The 22-Fr Melody valve delivery sheath being inserted through the incision. **(C)** Final appearance of the chest wall after closure. *Reprinted with permission from publisher: John Wiley and Sons* (44).

The base of the RV is accessed by a subxiphoid incision. This is followed by direct perventricular access through the RV initially with an angiocatheter, followed by sheath placement in the RVOT. Following sheath placement, a stiff wire, either a Amplatz Superstiff (Cordis, Johnson & Johnson, USA) or Lunderquist (Cook Medical, Bloomington, IN, USA), is secured in a distal branch pulmonary artery, and the RVOT is stented followed by transcatheter pulmonary valve placement. Once the valve has been deployed, follow-up angiography can be performed to confirm placement (Figure 4). Lastly, the sheath is removed, and the purse string suture is tied to close the RV, followed by subxiphoid incision closure (Figure 5).

### Potential Complications

This technique for pulmonary valve placement is feasible and with continued refinement and development can provide benefit to a range of patients with increased risk factors. There have been no post procedural complications reported. However, intra-procedural risk is present. In a recent case series by Gupta et al. (45), five patients with a mean weight 16.2 kg (range 4.7–28.1 kg) were treated with elective perventricular Melody valve placement *via* the subxiphoid approach. Technical success in this series was 100%. However, in two patients with absent pulmonary valve diagnoses, the stent migrated during advancement of the delivery sheath, requiring anchoring in the distal main or branch pulmonary artery, but without sequela. TV chordal injury also occurred in one patient, where TEE was not utilized. Therefore, this series demonstrates that the technique is feasible even in small-sized patients but with a need to minimize complications. First, the stent should be deployed during the same procedure, if possible, but if preexisting stenting has been performed, sheath advancement can be difficult and increases the risk of stent embolization. Hence, careful advancement of the delivery sheath is crucial, with proper securement of the wire. Additionally, the use of TEE can help to avoid TV entrapment during initial sheath advancement in the RVOT. Overall, the technique has an ongoing learning curve and with continued improvement may provide benefit to patients, young and old.

### Tetralogy of Fallot with Transannular Repair

The hybrid pulmonary valve replacement has also been trialed in previous anatomical scenarios once thought to be contraindications for transcatheter approach. In many patients with Tetralogy of Fallot, the initial surgical repair is performed with a transannular patch. Pulmonary regurgitation develops and chronically can leads to RV dysfunction, exercise intolerance, and cardiac arrhythmias (35). To address this, these patients eventually require pulmonary valve replacement. However, the complexity in this patient subset arises due to the chronic dilation of the RVOT, which leads to an unpredictable landing zone for transcatheter pulmonary valve placement. The percutaneous approach in these patients is difficult to perform but has been successfully reported by reducing the diameter of the RVOT either with implanting multiple stents (“Russian Doll Technique”) or overlapping stents in the branch pulmonary arteries and then implanting the valve (46–48). Yet, the problem still evades a strict percutaneous approach when the RVOT remains too large.

The hybrid approach allows an alternative strategy for manipulating the RVOT and MPA, prior to transcatheter pulmonary valve placement, and still avoids cardiopulmonary bypass (49). In a case in a 17-year-old male, through a midline sternotomy, a polytetrafluoroethylene graft (Gore-Tex, WL Gore & Associates Inc., Flagstaff, AZ, USA) was wrapped around the MPA to restrict the diameter to 22 mm (50). Following MPA restriction, a Melody valve was delivered through the perventricular approach over a 24-mm BIB balloon (NuMed Inc., Hopkinton, NY, USA). In an another recent study, MPA plication using longitudinal running sutures or mattress sutures was done to reduce the RVOT to 22–24 mm, followed by successful perventricular melody valve placement in three patients (51). Furthermore, 3D imaging modalities followed by use of hybrid strategy to place valves has been described (52).

### Pulmonary Atresia

In neonates with pulmonary atresia and anatomy favorable to biventricular repair, the initial intervention is focused on decompression of the RV and RVOT by creating either a surgical valvotomy or *via* transcatheter perforation and balloon valvuloplasty with or without stenting (53, 54). Several recent studies have demonstrated safety and feasibility of direct hybrid approach to perforate pulmonary valve (53–55).

## Hybrid Aortic Balloon Valvuloplasty

Congenital aortic stenosis in the neonate can present as an isolated lesion resulting in LV hypertrophy, and more significantly, severe systemic outflow obstruction, which can subsequently lead to sudden cardiac death (56). These patients often require multiple re-interventions throughout life due to their dysplastic aortic valve. To avoid initial open surgery in low weight infants, percutaneous balloon valvuloplasty was developed and does have respectable results but can be difficult in a low birth weight infant for several reasons (57–59). A hybrid technique to access the aortic valve directly through the ascending aorta has been described (60).

### Technique

In a recent study, this procedure was performed in 18 patients (mean weight: 4.6 ± 0.8 kg) (60). Patients were placed under general anesthesia, and a median sternotomy was performed with a purse string suture placed in the ascending aorta, and a wire was advanced and placed in the LV. An appropriate sized balloon was advanced for balloon aortic valvuloplasty. The results were fairly optimistic.

The majority of the aortic valvuloplasties, regardless of patient size, can be performed using transcatheter or carotid artery approach, and the feasibility of transaortic approach techniques will remain questionable.

## Conclusion

Hybrid techniques are thriving and will continue to do so as we continue to address our complex and small-sized patient population. Perhaps, the most critical ingredient of this technique is collaboration between the interventionalist and the surgeon in particular, and the involvement of the echocardiographer and the anesthesiologist in general.

As stated earlier, this review addresses only the common and some of the popular hybrid techniques in congenital heart disease. We have performed stent placement in the aortic arch at the time of bidirectional Glenn procedure in patients with coarctation of the aorta, closed complicated atrial septal defects using per-atrial technique, closed paravalvar mitral leak through access *via* the LV apex, and placed stents in branch pulmonary arteries intraoperatively. A detailed description of all the available techniques is beyond the scope of this manuscript.

## Author Contributions

All authors listed have made substantial, direct, and intellectual contribution to the work and approved it for publication.

## Conflict of Interest Statement

The authors declare that the research was conducted in the absence of any commercial or financial relationships that could be construed as a potential conflict of interest. The reviewer KD and handling Editor declared their shared affiliation, and the handling Editor states that the process nevertheless met the standards of a fair and objective review.
